# The Thirty-Third Long Fox Memorial Lecture: English Doctors from Smollett to Trollope
*It has been possible to print only a shortened version of the Lecture.


**Published:** 1946

**Authors:** E. Watson-Williams

**Affiliations:** Surgeon in charge of Ear, Nose and Throat Department, Bristol Royal Hospital. Lecturer in Diseases of the Ear, Nose and Throat, Bristol University


### The Bristol Medico-Chirurgical Journal

*" Scire est nescire, nisi id me*
*Scire alius sciret."*

AUTUMN, 1946.

### THE THIRTY-THIRD LONG FOX MEMORIAL LECTURE:

BY

E. Watson-Williams, Ch.M.,

*Surgeon in charge of Ear, Nose and Throat Department,*
*Bristol Royal Hospital.*
*Lecturer in Diseases of the Ear, Nose and Throat,*
*Bristol University.*

DELIVERED IN THE UNIVERSITY OF BRISTOL
ON WEDNESDAY, OCTOBER 18TH, 1944

ON

### ENGLISH DOCTORS FROM SMOLLETT TO TROLLOPE.*

What sort of people were the doctors in this country two hundred years ago and later ? How did they dress, speak and act, and how did their contemporaries regard them ? One way to answer these questions is to read the novels of the time : provided, of course, that we confine ourselves to those novels which attempt to portray contemporary life and manners, and which were accepted as such by the reading public of the day. Characterisation is sister to caricature : but we must not be too rigid—who, for example, would exclude the *Pickwick Papers* ?

* It has been possible to print only a shortened version of the Lecture.

### CHRONOLOGICAL TABLE, AUTHOR AND BOOK INDEX

(The numbers refer to pages in text. Index of Doctors p. 105)

| | | |
|---|---|---|
| Smallpox Inoculation, 1722 | 1719 | *Robinson Crusoe* (Defoe), *p*. 92. |
| GEORGE II, 1727 | 1726 | *Gulliver's Travels* (Swift), *p*. 92. |
| B.R.I. opened, 1737 | 1740 | *Pamela* (Richardson), *p*. 92. |
| London Corporation of Sur- | 1742 | *Joseph Andrews* (Fielding), *p*. 92. |
| geons: Second Jacobite Ris- | 1747 | *Clarissa* (Richardson), *p*. 92. |
| ing, 1745 | 1748 | *Roderick Random* (Smollett), *p*. 93. |
| *Midwifery* (Burton), 1751 *p*. 95. | 1749 | *Tom Jones* (Fielding), *p*. 93. |
| *Use of Water* (Smollett), 1752 | 1751 | *Peregrine Pickle* (Smollett), *p*. 94. |
| *p*. 94 | 1752 | *Ferdinand* (Smollett), *p*. 94. |
| *Midwifery* (Smellie), 1753 *p*. 94. | 1760 | *Tristram Shandy* (Sterne), *p*. 95. |
| Johnson's Dictionary, 1755 | 1761 | *Vicar of Wakefield* (Goldsmith). |
| GEORGE III, 1760 | 1762 | *Sir Launcelot Greaves* (Smollett), *p*. 94. |
| Auenbrügger's Auscultation | 1771 | *Humphrey Clinker* (Smollett), *p*. 95. |
| R.C.S. founded .. .. | 1778 | *Evelina* (Burney), *p*. 95. |
| French Revolution, 1789 | 1782 | *Cecilia* (Burney), *p*. 95. |
| Vaccination, Jenner .. | 1796 | *Camilla* (Burney). |
| Laughing Gas, Davy, 1800 | 1801 | *Belinda* (Edgeworth). |
| Trafalgar, 1805 | 1811 | *Sense and Sensibility* (Austen), *p*. 96. |
| | 1813 | *Pride and Prejudice* (Austen), *p*. 96. |
| Waterloo } 1815 | 1814 | *Mansfield Park* (Austen). |
| Apothecaries Act } 1815 | 1816 | *Emma* (Austen), *p*. 96; *Headlong Hall* (Peacock). |
| GEORGE IV, 1820 | 1818 | *Persuasion* (Austen). |
| Stethoscope .. .. | 1822 | *Opium Eater* (De Quincey), *p*. 96. |
| *Lancet* founded .. .. | 1824 | *Our Village* (Mitford), *p*. 92. |
| WILLIAM IV } 1830 | 1826 | *Vivian Grey* (Disraeli). |
| First Railway } 1830 | 1831 | *Jorrocks* (Surtees), *p*. 98. |
| Anatomy Act } 1832 | 1832 | *Eugene Aram* (Lytton). |
| B.M.A. founded } 1832 | 1836 | *Pickwick* (Dickens), *p*. 98. |
| VICTORIA, 1837 .. .. | 1837 | *Oliver Twist* (Dickens), *p*. 99. |
| | 1838 | *Nicholas Nickleby* (Dickens), *p*. 99. |

Let us first give a short glance at the conditions of professional life. In 1740 the medical profession was in a state of flux. The influence of trade guilds was waning, their regulations being regarded as old-fashioned and irksome restraints on individual enterprise. In Bristol, for example, the last public appearance of the Barber-Surgeons was in 1742, and the remaining members mortgaged their hall in 1750 [Parker, 1912]. The Surgeons were by this time almost entirely separated from the barbers, though barbers who "bled" and drew teeth lasted on until the end of the century [Harsant, 1899].

Until the Medical Act of 1858 the control of practice (surgery included) was in London and for seven miles round vested in the Royal College of Physicians: in other large cities, in the hands of the local Barber-Surgeons' Guild. In London the Corporation of Surgeons licensed surgeons only. In smaller towns and the countryside, by an Act of Henry VIII, each Bishop was required to regulate practice, to license practitioners (of course, after proper training and examination) and midwives, and to prosecute irregular practitioners and quacks: Latimer gives the date of the last licence issued

CHRONOLOGICAL TABLE. AUTHOR AND BOOK INDEX—contd.
(The numbers refer to pages in text)

| | | |
|---|---|---|
| *Punch* begins .. .. | 1841 | *Old Curiosity Shop* (Dickens), *p.* 99. |
| | 1843 | *Handley Cross* (Surtees), *p.* 100; *Martin Chuzzlewit* (Dickens), *p.* 99. |
| | 1845 | *Sybil* (Disraeli). |
| Anæsthesia, 1846 .. .. | 1847 | *Jane Eyre* (C. Brontë), *p.* 100; *Vanity Fair* (Thackeray), *p.* 101. |
| | 1848 | *Pendennis* (Thackeray), *p.* 100. |
| | 1849 | *David Copperfield* (Dickens), *p.* 99; *Caxtons* (Lytton). |
| *Pathology* (Virchow) .. | 1850 | *Alton Locke* (Kingsley). |
| Great Exhibition .. | 1851 | *Lavengro* (Borrow). |
| | 1852 | *Bleak House* (Dickens), *p.* 99; *Esmond* (Thackeray). |
| | 1853 | *Cranford* (Gaskell), *p.* 101. *The Newcomes* (Thackeray) *p.* 100 |
| | 1855 | *The Warden* (Trollope), *p.* 102; *Westward Ho!* (Kingsley). |
| | 1857 | *Barchester Towers* (Trollope), *p.* 103; *Little Dorrit* (Dickens), *p.* 99. |
| Medical Act .. .. | 1858 | *Doctor Thorne* (Trollope), *p.* 103; *Clerical Life* (Eliot), *p.* 96. |
| | 1860 | *Woman in White* (Collins). |
| | 1861 | *Framley Parsonage* (Trollope), *p.* 104; *Silas Marner* (Eliot), *p.* 96. |
| Sphygmograph (Marey) | 1863 | *Hard Cash* (Reade) *p.* 102; *Water Babies* (Kingsley). |
| | 1864 | *Small House at Allington* (Trollope), *p.* 104. |
| | 1865 | *Alice in Wonderland* (Carroll). |
| Antiseptic Method (Lister) | 1866 | *Felix Holt* (Eliot), *p.* 97. |
| | 1867 | *Last Chronicle of Barset* (Trollope), *p.* 104. |
| | 1871 | *Middlemarch* (Eliot), *p.* 97. |

by the Bishop of Bristol as 1745. The Corporation and Bishops' licences were of local validity. But in addition the Universities could license graduates to practise anywhere in the kingdom, and it appears that a foreign degree was recognised as a title to practise.

"Physicians held their heads very high. They were some of the best educated men of that day. They had all a good knowledge of the classics . . . were very particular in their dress and deportment, and were easily distinguished from the common herd." [Harsant.] They were all University graduates, and tended to look down upon humbler ranks in the profession.

Surgeons were not, as a rule, graduates: in the country they were often apothecaries as well as surgeons. Their training was by an apprenticeship, properly for seven years, tested by examinations by the local guild. A period of hospital training was usual. Even as late as 1812, (Sir) Charles Hastings, for example, having completed apprenticeship and hospital training, was appointed house surgeon to Worcester Infirmary with no degree or diploma: he went later to Edinburgh, and graduated in 1818.

General practice was mainly in the hands of apothecaries.

Their training was by apprenticeship, usually for five years: they were not paid by fees, but only for medicines supplied—but made up for this by supplying enormous quantities, each dose in a separate phial. Many of them, especially in towns towards the end of the eighteenth century, became very rich: until the public found it was cheaper to get medicines from a druggist. The Apothecaries Act (1815) made it legal for them to charge for advice.

In addition, quacks were numerous: not merely the sellers of secret remedies or the elixir of life; but anyone with sufficient impudence, who had failed to make a living in his own calling, could set up as a doctor—provided he avoided the few places where the local guilds were still active.

There was certainly no lack of medical men. In the novels of Fielding and Smollett, for example, brawls are frequent; there is never any delay in securing professional care for broken heads, and even in the country a consultation with a physician was not difficult. In Bristol we find:—

| | 1754 [Harsant] | 1794 [Matthews] |
|---|---|---|
| Doctors of Medicine ... ... ... | 5 | 14 |
| Surgeons ... ... ... ... | 19 | 26* |
| Barber-Surgeons ... ... ... | 13 | |
| Apothecaries ... ... ... ... | 29 | 34* |
| Druggists ... ... ... ... | | 4 |
| Population [Latimer] ... | 45,000 | 65,000 |

The 1841 Census gave 33,339 persons as practising medicine; the (unofficial) *Medical Directory* of 1853 shows only 11,808 as qualified (by examination): the proportion of practitioners to population was evidently as high just before the passing of the first Medical Act as in 1939. [History of B.M.A.]

The novel is a comparatively late form. Collections of stories range from the *Arabian Nights*, *Decameron* and *Our Village* to *Victoria 4.30*: their influence persists in the novel in "inclusions," such as the *Memoirs of a Lady of Fashion* in Smollett's *Peregrine Pickle* and the Bagman's and other stories in *Pickwick*. The novel of incident developed directly from tales of travel, etc., such as *Robinson Crusoe* and *Gulliver's Travels*; Lemuel Gulliver, by the way, is described as "First a Surgeon and then a Captain of Several Ships."

The novel of character began with SAMUEL RICHARDSON (1689–1761), a printer, "the acknowledged creator of the English Novel." His first novel, *Pamela*, is irrelevant to our present purpose, but inspired Fielding to his skit of it, *Joseph Andrews*. *Clarissa* appeared

* Three surgeons were "surgeon and apothecary," one surgeon and one apothecary were also "man-midwife," one surgeon was also "oculist." Bristol Hotwells was a noted health resort throughout the eighteenth century, which may explain the high proportion of consultants [Griffiths].

in 1747. It is very long—nearly a million words, about four times the size of *Pickwick*, and the references to doctors are unimportant : there are Mr. Goddard the apothecary and Dr. H. whom he calls in consultation.

HENRY FIELDING (1707–54), the Westminster Magistrate, may be considered next, although the novel by which he is remembered, *Tom Jones*, was published (1749) later than Smollett's first work. It contains numerous sarcastic references to medicine.

Dr. Blifil had the misfortune of losing the advantage of great talents by the obstinacy of a father, who would breed him to a profession he disliked. He had been obliged to study physic, or rather, to say he studied it. Unfortunately for him, the doctor was master of almost every other science but that by which he was to get his bread. The consequence was that at the age of forty the doctor had no bread to eat.

[Tom Jones joins a company of soldiers going north to repel the Jacobite rebels, and is hit on the head in a brawl. The landlady of the inn renders first-aid, mainly brandy.] Soon afterwards arrived the surgeon, who having viewed the wound, having shaken his head and blamed everything that was done, ordered the patient to bed. The lieutenant enquired, " Sir, I hope his skull is not fractured." " Fractures are not always the most dangerous symptoms. Contusions and lacerations are often attended with worse phenomena and with more fatal consequences than fractures. People who know nothing of the matter conclude if the skull is not fractured all is well . . . Symptoms are not always regular or constant. I have known very unfavourable symptoms in the morning change to favourable at noon and return to unfavourable at night. I was, I remember, once called to a patient who had received a violent contusion in his tibia, by which the external cutis was lacerated, so that there was a profuse sanguinary discharge ; and . . . the os or bone very plainly appeared through the vulnus or wound " [continuing with a verbose clinical lecture on treatment of a cut shin. He then prescribes for Jones ; but hearing next day that his patient has no money, declines to attend further.

" Little Benjamin " (Partridge) is called to shave Jones and proves to be a merry wag. He had been a schoolmaster, and is full of classical tags ; but that failing he set up as a barber and is locally reputed a skilful surgeon. He goes out, puts on his coat, and returns with a dignified air to attend Jones in the latter capacity—and tells him the cut is trivial and he may get up ! He then accompanies Jones in his travels and is eventually re-established as a schoolmaster, but gives no further evidence of surgical abilities].

TOBIAS GEORGE SMOLLETT (1721–71) was a fellow-student with William Hunter at Glasgow, and later apprenticed to Dr. John Gordon, whom he eulogises in *Humphrey Clinker*. He obtained an appointment as Surgeon's Mate aboard the *Cumberland* in Vernon's expedition to Cartagena (1740) : much of *Roderick Random* is autobiography. He later practised in Downing Street, in Bath, and in Chelsea : or rather, resided in these places, for like Goldsmith (and some of our contemporaries) he found literature more attractive and much more remunerative. He published a treatise on *The External use of Water* (1752), and edited several works, including Smellie's *Midwifery.*

In *Roderick Random* (1748) we find many medical portraits.

The apothecary, Mr. Launcelot Crab, was aged fifty, about five feet high and ten round the belly : his face capacious as the full moon and much of the complexion of a mulberry : his nose, resembling a powder-horn, was swelled to an enormous size, and studded all over with carbuncles : and his little grey eyes reflected the light in such an oblique manner that, while he looked a person full in the face, one would have imagined he was admiring the buckle of his shoe.

Mr. Lavement was a little, odd, withered man with a forehead about an inch high, a nose turned up at the end, large cheek-bones that helped to form a pit for his little grey eyes, a great bag of loose skin hanging down on each side in wrinkles like the alforjas of a baboon : and a mouth so accustomed to that contraction which produces grinning, that he could not pronounce a syllable without discovering the remains of his teeth, which consisted of four yellow fangs not improperly by anatomists called canines . . . To see him without the least hesitation make up a physician's prescription, though he had not in his shop one medicine mentioned in it . . . a hundred costly preparations were produced in an instant from the cheapest and coarsest drugs. [Description of examination at the College of Surgeons.]

[Aboard the *Thunder*, Dr. Atkins is surgeon, and Mr. Thompson the colourless second mate.] Mr. Morgan, the first mate, was a short thick man with a face garnished with pimples, a snub nose turned up at the end, an excessive wide mouth and little fiery eyes surrounded by innumerable wrinkles.

[He was very Welsh, proud and irrascible ; and appears throughout the first half of the book : descriptions of sick bay and sick parade. Other surgeons are] Mr. Simper, a young man gaily dressed, of very delicate complexion and Dr. Mackshane, grossly ignorant, intolerably assuming, false, vindictive and unforgiving : a merciless tyrant.

*Peregrine Pickle* (1751) contains a lampoon of Dr. Akenside travelling in France : and an account of Smollett's club for authors, described also in *Humphrey Clinker.* Pickle is ill and is attended by a member of the club, in whom we can recognise Dr. Oliver Goldsmith (1728–74) : Dr. Goldsmith's novels have no medical references. In Chapter LXXXVII we find the plot of *Pygmalion.*

*Ferdinand, Count Fathom* (1752) is the account of a rogue who, among other occupations practised medicine in Bristol, London and Tunbridge Wells : with several sketches of doctors in those places. Ferdinand appears again in *Humphrey Clinker* as an apothecary called Grieve.

*Sir Launcelot Greaves* (1762) gives us " Mr. Fillet, a country practitioner in surgery and midwifery, a man of some education and a great deal of experience, shrewd and sensible": Mr. Ferret, a lawyer's clerk, peddling the "Elixir of Life" in a country town: the oft-quoted scene between apothecary and physician when Crabshaw is ill: and another physician in a mad-house where Sir Launcelot is confined.

*Humphrey Clinker* (1771), in addition to the references already mentioned, provides accounts of Bristol and Bath spas: "Dr. L., who is come to ply at the Hot Well for patients" (Dr. D. W. Linden, [Griffiths]): and an account of preparations for trephining, which the patient refused.

LAWRENCE STERNE (1713–68) gives us in *Tristram Shandy* (1760) a caricature of Dr. John Burton, who published his *System of Midwifery* in 1751; though the events are supposed to occur in 1713.

Imagine to yourself the little, squat, uncourtly figure of a Doctor Slop, about four feet and a half perpendicular height, with a breadth of back and a sesquipedality of belly which might have done honour to a sergeant in the horse guards. Such were the outlines of Dr. Slop's figure, which . . . may as certainly be caricatured and conveyed to the mind by three strokes as by three hundred. Imagine such a one coming slowly along, foot by foot, waddling through the dirt upon a little diminutive pony. He was advancing to Shandy Hall and had approached to within sixty yards of it, when Obadiah and his coach-horse turned the corner, rapid, furious—pop—full upon him. What could he do? He crossed himself—Pugh—(but the Doctor, Sir, was a Papist)—he had better have kept hold of the pommel. Without waiting for Obadiah's onset he left his pony to its destiny, tumbling off it diagonally in the mire. Never was a Doctor Slop so beluted and transubstantiated. [He sends off Obadiah to fetch his green bag of obstetric forceps: takes up Mr. Shandy for calling him the man-midwife—"Accoucheur, if you please." And when the bag is brought, delivers a lecture on obstetric advances, with a demonstration of the forceps in which he crushes Uncle Toby's hand: and goes upstairs to break Tristram's nose by faulty application of the forceps!]

FANNY BURNEY (Madame D'Arblay, 1752–1840), Dr. Johnson's "little character-monger," began to write at the age of 10. In *Cecilia* (1782) we meet Dr. Lyster.

A humane and excellent physician and a man of sound judgement. "Pray, sir, have patience. Mr. Mortimer and I will have some discourse together presently. Meantime, let us all sit down and behave like Christians. I never talk of my art before company. 'Tis hard you won't let me be a gentleman at large for two minutes."

[Later he tells Mrs. Delville her son needs only ordinary care and then a stay at Bristol for convalescence. Fanny Burney is fond of sending her characters to Bristol—Mrs. Delville is consigned there later, when she breaks a blood vessel, and much of *Evelina* (1778) is placed in Clifton and the Hot Wells. Dr. Lyster appears several times, usually to tell various members of the family to keep calm—very necessary advice. The book closes with a commentary on Pride and Prejudice which supplied the title to Jane Austen's second novel (1813)].

JANE AUSTEN (1775–1817) mentions doctors only casually in some of her books. In *Sense and Sensibility* (1811) there is Mr. Harris, quietly self-confident and rejecting the appeals for a second opinion. But Jane Austen hardly ever *describes*: her characters are built up by a touch here, another there, a word, an act. In *Emma* (1816) we hear constantly of Mr. Perry, sufficiently assiduous to please even valetudinarian Mr. Woodhouse.

[When Emma had the measles he attended four times a day. He regularly confirms all Mr. Woodhouse's fussy precautions about diet, draughts and wet feet. He is happily married—" I suppose there never was a happier or a better couple than Mr. and Mrs. Perry." And his wife has persuaded him to set up a carriage as he is getting too old to do his rounds on horseback.] " Poor Perry is bilious, and he has not time to take care of himself. He tells me he has not time to take care of himself—which is very sad—but he is always wanted all around the country. I suppose there is not a man in such practice anywhere. But then, there is not so clever a man anywhere. I have a great regard for Mr. Perry."

THOMAS DE QUINCEY (1785–1859), begins *The Confessions of an English Opium-eater* (1822), with his boyhood twenty years earlier.

This person was not a physician, who would of course have expected the ordinary fee of a guinea for every visit: nor a surgeon, but simply an apothecary . . . He was a comatose old gentleman, rich beyond all his needs, careless of his practice and standing under that painful necessity (which prohibits fees to apothecaries) of seeking his remuneration in excessive doses of medicine. Me, however, out of pure idleness, he forebore to plague with any variety of medicines. With sublime simplicity he confined himself to one horrid mixture that must have suggested itself when prescribing for a tiger . . . I begged to know if his art had not amongst its reputed infinity of resources any less abominable. " None whatever," he replied. Exceedingly kind he was: insisted on my drinking tea with his really amiable daughters: but continued at intervals to repeat, " None whatever—none whatever —whatever—ever—ver—er . . ."

GEORGE ELIOT (Mary Ann Evans, 1819–80) describes many scenes of her childhood and youth, spent with her father, a Warwickshire land agent. In *Scenes from Clerical Life* (1857), we find Mr. Pilgrim and Mr. Pratt, friendly rivals, united in preventing any third practitioner settling in Milby. *Silas Marner* (1861):

Dr. Kimble (country apothecaries in old days enjoyed that title without authority or diploma), a thin and agile man, [was spending Christmas at the home of Squire Cass, his brother-in-law, and talking of his experiences in London Hospitals thirty years before]. He was welcomed everywhere as Doctor by hereditary right—not one of these miserable apothecaries who canvass for practice in strange neighbourhoods and spend all their income starving their one horse ; but a man of substance, able to keep an extravagant table like the best of his patients. Time out of mind the Raveloe doctor had been a Kimble, and it was difficult to contemplate firmly the melancholy fact that the actual Kimble had no son. [He is sent for and turns out] coming from the card-room in some bitterness at the interruption, but drilled by the long habit of his profession into obedience to unpleasant calls, even when he was hardly sober.

*Felix Holt* (1866) [is the son of a quack medicine maker]. I was 'prentice for five miserable years to a stupid brute of a country apothecary. . . . I know the cathartic pills are a drastic compound which may be as bad as poison to half the people who swallow them ; that the elixir is an absurd farrago of a dozen incompatible things ; and that the cancer cure might as well be bottled ditch-water. [So he gives up medicine and turns reformer.]

*Middlemarch* (1871) relates to 1829. The low-church radical banker Bulstrode has just founded the new Hospital in opposition to the old Infirmary supported by the county, mercantile and high-church party :

[The new doctor, Lydgate] "is tall, dark, clever. I am told he is wonderfully clever : he certainly looks it. He is a gentleman ; he is one of the Lydgates of Northumberland, really well connected. One does not expect it of a practitioner of that kind. For my part I like a medical man more on a footing with the servants—they are often all the cleverer. Poor Hicks was coarse and butcher-like, but he knew my constitution."

Mr. Lydgate had the medical accomplishment of looking perfectly grave whatever nonsense was talked to him. He confirmed Lady Chettam's view that her own constitution was peculiar—more peculiar than others.

" I think he is likely to be first-rate : studied in Paris, has ideas ; wants to raise the profession." " That's all very fine. If you want him to try experiments on your hospital patients, and kill a few people for charity, I have no objection. But I'm not going to hand money out of my purse to have experiments tried on me."

There was a general impression that Lydgate was not altogether a common country doctor. Each lady who saw medical truth in Wrench and the strengthening treatment regarded Toller and the lowering system as perdition. No one supposed Mr. Lydgate could know as much as Dr. Sprague or Dr. Minchin, the two physicians, who alone could offer hope when danger was extreme and when the smallest hope was worth a guinea. [Lydgate uses a stethoscope : causes offence to colleagues and patients by charging for advice and not dispensing medicine ; people are upset by rumours of autopsies. Mr. Gambit, with an extensive midwifery practice, never charges for advice ; only for medicine which he supplies freely. There are references to the prosecution of Dr. Edward Harrison in 1828 for practising without a licence from the College of Physicians, and to the subversive views of Wakley of the *Lancet*.]

CHARLES JOHN HUFFAM DICKENS (1812–70) was already beginning to be known as "Boz" when the success of the *Jorrocks* sketches sent Chapman and Hall looking for an author to write humorous sporting articles, and *Pickwick* (1836), to condense its absurd and verbose title, was the result.

Dr. Slammer, surgeon to the Ninety-seventh, was a little fat man with a ring of upright black hair round his head and an extensive bald plain on the top of it. He took snuff with everybody, chatted with everybody, laughed, danced, made jokes, played whist, did everything, and was everywhere. [He challenges Mr. Winkle to a duel and brings Dr. Payne "a portly personage in a braided surtout," who is very disappointed at the duel not taking place.]*

Mr. Benjamin Allen† [of St. Thomas's Hospital,] was a coarse, stout, thickset young man with black hair cut rather short and a white face cut rather long. Hewore spectacles and a white neckerchief. Below his single-breasted black surtout, buttoned up to his chin, appeared pepper-and salt-coloured legs terminating in imperfectly polished boots. . . . He presented altogether rather a mildewy appearance. Mr. Bob Sawyer [of Guy's] was habited in a coarse blue coat which, without being either a great-coat or a surtout, partook of the nature and qualities of both. He had about him that sort of slovenly smartness and swaggering gait peculiar to young gentlemen who smoke in the streets by day, and call waiters by their Christian names. He wore a pair of plaid trousers and a large rough double-breasted waistcoat. [And later gives a party at his lodgings in Lant Street, Borough. We meet Jack Hopkins, Bart.'s, Noddy, Gunter and other medical students. Like the modern medical student he delights in describing the exciting "case" or operation, to astonish his friends. In Chapter XXXVIII Mr. Winkle loses his way in Bristol in the tangle of small streets between College Green and the road to Clifton, then, of course, via Lower Clifton Hill; and enquiring at the Surgery of Sawyer, late Nockemorff,‡ discovers the two friends established in practice.] "I *passed* soon after that precious party and my friends came down with the needful for this business, so I put on a black suit of clothes and a pair of spectacles, and came here, to look as solemn as I could. . . . Half the drawers have got nothing in 'em and the other half don't open. Hardly anything real in the shop but the leeches, and *they* are second-hand. [Chapter XLVIII.] It's wonderful how the poor people patronise me: they knock me up at all hours of the night. . . . It's very gratifying, only not quite so much

* Pugh suggests that Dr. Slammer bore a colourable resemblance to Dickens's uncle Dr. Lamert: Hugh Kingsmill recounts that he was told by the doctor's daughter in her old age that the original was Dr. Parkes, surgeon to the 79th Regiment, stationed at Chatham when Dickens was a small boy.

† Dr. Benjamin Allen published *The Natural History of the Mineral Waters of Britain* in 1711: with a eulogy of the Bristol Well [Griffiths].

‡ Mr. B. Wright of Druid Stoke tells me that he used to know Michael Hy., Clark, Surveyor, who died about 1910: the latter was brought up as a child in his father's house at the foot of Park Street, which his father always maintained was the original of the surgery. Michael Clark, druggist, appears in *Bristol Directories*, 1832–1854, at 65 Park Street (bottom of Park Street, before the bridge was built over Frogmore Street, on the right side looking up).

as the confidence of patients with a shilling or two to spare would be. This business was capitally described in the advertisement: it's a practice, a very extensive practice—and that's all."

Bob Sawyer is rather a pathetic figure—an orphan, poor, in wretched lodgings in Lant Street (where Dickens lodged when his father was in the Marshalsea) and in debt to a very unpleasant landlady. His clothes were shabby, but probably not from choice: and smoking in the street cannot even then have been a serious vice. But he is always cheerful, very hospitable, a staunch friend to the surly Allen, and, as the skating scene shows a good sportsman and good-tempered. He got drunk, but so did everyone else in *Pickwick*: he worked at least enough to qualify in due course, was then put into a poor working-class practice and rapidly established a large (if non-paying) connection. When he went bankrupt he and Allen went to Bengal and "had the yellow fever fourteen times"—the only cases of this disease recorded in India!

Dickens's other doctors are not very interesting: they appear mostly for obstetric reasons: as Mr. Lumbey, the Kenwigs's doctor in *Nicholas Nickleby* (1838) and as in the horrid accouchment in the Marshalsea (*Little Dorrit* 1857).

Mr. Losberne (*Oliver Twist* 1837) "a surgeon known for miles round as 'the doctor' had grown fat more with good humour than good living: and was as kind and hearty, and withal as eccentric an old bachelor as will be found. . . . The excellent doctor had never acted upon anything but impulse and it was no bad compliment . . . that he enjoyed the warmest respect and esteem from all."

The doctor who is called to little Nell (*Old Curiosity Shop*, 1841) was "a red-nosed gentleman with a great bunch of seals dangling below a waistcoat of ribbed black satin."

In *Martin Chuzzlewit* (1843) [we meet, beside Mr. Lewsome the apothecary and Mrs. Gamp, the unctuous Mr. Jobling, M.R.C.S., surgeon to the Anglo-Bengalee Loan and Life Insurance Company]. "We know a few secrets of nature in our profession, sir, of course we do. We study for that. We pass the Hall and the College for that. . . ." He had a portentously sagacious chin and a pompous voice . . . could shake his head and rub his hands . . better than any man alive: he had a way of smacking his lips and saying "Ah" which gave patients the greatest confidence. His female patients could never praise him too highly.

Dr. Parker Peps (*Dombey and Son*, 1847), one of the court physicians, [could never remember when he attended a commoner that his patient was not a duchess; Mr. Pilkins, the family practitioner, knew how difficult it must be for such a man].

Mr. Chillip (*David Copperfield*, 1849) was the meekest of his sex, the mildest of little men.

Mr. Bayham Badger (*Bleak House*, 1852) had a good practice in Chelsea and attended a large public institution besides. He was a pink, fresh-faced, crisp-looking gentlemen, with a weak voice, light hair and surprised eyes, some years younger than his wife—who constantly announced that he was her third husband. [Richard Carstone was apprenticed to him. He thought highly of Mr. Woodcourt because "his heart is in his work." Mr. Woodcourt, the ship's surgeon, displayed great courage and enterprise during a shipwreck. He attends a woman in the street. While he was thus employed he says:] "And so your husband is a brickmaker ?" "How do you know that, Sir ?" "Why, I suppose so, from the colour of the clay upon your bag and on your dress. And I know that brickmakers . . . are often cruel to their wives."*

ROBERT SMITH SURTEES (1803–64) started the *New Sporting Magazine* in which *Jorrocks* first appeared : *Handley Cross* (1843) introduces the rival doctors :

Mr. Roger Swizzle was in height five feet eight inches and forty years of age. He had a fat red face with little twinkling black eyes set high in his forehead. . . . A good-natured smile hovered round the dimples of his cheeks which set a patient at ease in a moment. [He set up in Handley Cross and recommends early hours, good plain food and plenty of it, no side dishes : affects the bluff and hearty manner and makes a great success.]

A thriving trade soon brings competition : another patientless doctor determined to try his luck. Dr. Sebastian Mello devoted himself to the serious. He was about forty, but a fair complexion, flowing sandy locks and a slight figure made him look ten years younger. He had a Grecian face, with blue eyes and good teeth. His black suit fitted without a wrinkle and his thin dress-boots shone with patent polish : turned-back cambric wrist-bands displayed the snowy whiteness of his hand and set off his massive antique ring. He drove about in a claret-coloured fly. . . . He kept a large quarto volume into which he entered each case and its daily symptoms, writing Latin prescriptions for the chemists . . . and conducted a lively controversy with himself in the London papers. [It turns out eventually that he has no qualifications !]

CHARLOTTE BRONTE ("Currer Bell" 1816–55) in *Jane Eyre* (1847) introduces "Mr. Lloyd, an apothecary sometimes called in by Mrs. Reed when the servants were ailing. For herself and the children she employed a physician." He examines Jane at Lowood Charity Institution, and shows his intelligence by getting rid of the nurse, so that Jane can speak without fear. At Thornfield we meet Mr. Bates the apothecary and Mr. Carter the surgeon.

WILLIAM MAKEPEACE THACKERAY (1811–63) describes several doctors in *Pendennis* (1848) : the novel is dedicated to Dr. John Elliotson (University College Hospital), an enthusiast in Medical Magnetism, and the original of Dr. Goodenough who attends Pendennis when he has typhus (and Col. Newcome in *The Newcomes* 1852).

* ("Elementary, my dear Watson !" Ed.)

Pendennis (senior) had a Cornish pedigree which carried the Pendennises up to the time of the Druids : they were related to all the great families of Wales. He had had a piece of University education . . . but in his second year his father died insolvent. He was apprenticed to a London apothecary of low family, but quickly after his apprenticeship parted from the coarse-minded practitioner and set up for himself in Bath, where he exercised the profession of apothecary and surgeon : and not only attended gentlemen in their sick-rooms and ladies at the most interesting periods of their lives, but would sell a brown paper plaster to a farmer's wife across the counter, or vend tooth brushes and hair powder. . . . First his humble shop became a smart one ; then he discarded the selling of tooth brushes as unworthy of a gentleman ; then he shut up the shop altogether . . . and before her exit from this world his mother had the happiness of seeing her beloved John step into a close carriage of his own, a one-horse carriage it is true, but with the arms of the family of Pendennis handsomely emblazoned on the panel. . . . A little gentleman who rapped his teeth and smiled artificially, who was laboriously polite to the butler as he slid upstairs into the drawing-room, and perfectly civil to the lady's maid who waited at the bedroom door ; for whom Lady Rockminster used to ring as for a servant.

Mr. Samuel Huxter (Bart.'s), a young man in a large white coat with a red neckcloth, over which a dingy shirt collar was turned, with a large pin of bullion or some other metal and an imaginative waistcoat with exceedingly fanciful glass buttons, and loud trousers. [He is cheerful and convivial], the life and soul of the circle . . . his affableness and social turn made him quickly intimate with persons in whose society he fell. [Later Mr. Foker introduces him as] " my excellent friend and body-surgeon, Mr. Samuel Huxter, M.R.C.S."

Old Mr. Huxter was in great good humour : " I can afford to give myself a lark, sir. . . . A man has to take his business where he finds it, and I succeeded to that of my father." The old surgeon was delighted to speak to a coroneted carriage in the midst of the full Strand : he ran out bowing and smiling. " What were her Ladyship's symptoms ? Should he meet her Ladyship's usual medical attendant ? " [Old Major Pendennis refers to Sir Derby Oak, the celebrated Physician ; and to Mr. Firmin " man of good family—Firmin might go anywhere."]

In *Vanity Fair* (1847), we find Dr. Pestler, a flourishing lady's physician, Mr. Linton his assistant, Mr. Clump the apothecary and Dr. Squills.

MRS. GASKELL (Elizabeth Cleghorn Stevenson, 1810–65) is best known for *Cranford* (1853) : the story refers to about 1835.

Mr. Hoggins was the Cranford doctor. We disliked the name and considered it coarse, but as Miss Jenkyns said, if he changed it to Piggins it would not be much better. A man whom the Honourable Mrs. Jamieson had tabooed as vulgar, and inadmissable to Cranford society ; not merely on account of his name, but because of his voice, his complexion, his boots smelling of the stable and himself smelling of drugs. Tradition went that his boots were the identical pair in which he had first set out on his rounds in Cranford twenty-five years ago : only they had been new-pieced high and low, top and bottom, heel and sole, more times than anyone could tell. (It is announced that he is to marry Lady Glenmire.) " My Lady will have to come down to many a want of refinement. Mr. Hoggins sups on bread and cheese every night. . . ." " I don't know : Mr. Hoggins is rich and very pleasant looking, and very good-tempered and kind-hearted. Mr. Hoggins is really a very personable man, and as for his manners, why, if they are not very polished, I have known people. . . ."

CHARLES READE (1814–84) in *Hard Cash* (1863), introduces no less than fourteen doctors !

Mr. Osmond, a consulting surgeon, bore a high reputation in Barkington. He came, and proved too plump for that complete elegance she would have desired : but had a soft hand, a gentle touch and a subdued manner . . . and told Mrs. Dodd it was a case of excessive sensibility.

" Dr. Short . . . I have heard of him : I have even met him in society, a most refined person." He was six feet two, wonderfully thin, livid and gentleman-like—fine long head, keen eye, lantern jaws. " Dr. Osmond—Osmond ? I do not know that name in medicine. Ah ! you mean Mr. Osmond, a surgeon. I do not know a more estimable person—in his grade of the profession. And so he gives his opinion in medical cases, does he ? " [To London to " the morning levée of Sir William Best," who uses a stethoscope and diagnoses heart disease. Julia wants to see a lady doctor, but is taken to Dr. Chalmers " the Court Physician," and to Dr. Kenyon.]

Dr. Sampson was himself afflicted with what I shall venture to call a mental ailment, to wit, a furious intolerance of other men's opinions. [He always orders the exact opposite of the usual, talks a clipped Yorkshire dialect, extols Chronothermal Medicine, of which he is the inventor.] He leapt upon the woman with the agility of a mountain cat and chloroformed her with all his might ! [The others are Drs. Stephenson, Wycherley, Speers, Wolf, Eskell, Terry, Phillips and an asylum doctor.]

ANTHONY TROLLOPE (1815–82) wrote sixty novels. The " Barchester " six are still popular : in these the same characters appear and reappear. *The Warden* (1855) contains an attack on Dickens as " Mr. Sentiment."

[John Bold, a young surgeon, studied in London and on his father's death] settled himself at Barchester to look after his property as well as the bones and bodies of such of his neighbours as would call upon him. He therefore put up a large brass plate with " John Bold, Surgeon " on it, to the great disgust of the nine practitioners who were already trying to get a living out of the Bishop, Dean and Canons : and began housekeeping with the aid of his sister. Though he has now been three years in Barchester he has not taken three fees. Though a clever man, who would with practice be a clever surgeon, having enough to live on he has declined to subject himself to what he calls the drudgery of his profession. His passion is the reform of abuses—

State abuses, Church abuses, corporation abuses, abuses in medical practice. . . . He is brave, eager and amusing ; well made and good-looking, young and enterprising.

[In *Barchester Towers* (1857) Bold is dead.] I never thought him worthy of the wife he had won. . . . That arrogance of thought, unsustained by first-rate abilities, that attempt at being better than his neighbours. . . .

Sir Lambda Mew-New and Sir Omicron Pie, the two great London doctors, now came down for the fifth time and declared, shaking their learned heads, that another week of life was impossible. . . . They had been thrice wrong and might be thrice wrong again. [Dr. Fillgrave and Mr. Rerechild appear].

*Doctor Thorne* (1858), became settled for life in the little village of Greshamsbury. As was then the wont with country practitioners . . . he added the business of a dispensing apothecary to that of a physician. In doing so he was of course much reviled. Many people declared that he could not truly be a doctor : and his brethren in the art living around him, though they knew that his diplomas and degrees were all in order, rather countenanced the report. . . . They considered him *de trop*. Greshamsbury was only fifteen miles from Barchester where there was a regular depot of medical skill, and but eight from Silverbridge, where Dr. Century had been in residence forty years. . . . Dr. Thorne had no appreciation of the dignity of a learned profession. He might constantly be seen compounding medicines in the shop—not making experiments philosophically in *materia medica* . . . but positively putting together common powders or spreading vulgar ointments for agricultural ailments. A man of this sort was not fit society for Dr. Fillgrave . . . Dr. Fillgrave declined to meet Dr. Thorne in consultation.

Dr. Thorne understood his business and was willing to labour at it with energy. But he was proud of his descent, brusque, authoritative—to trifling ailments often too brusque. His wants were few : he had a few bottles of good wine and occasionally asked a brother bachelor to take a chop with him.

Dr. Fillgrave was not a tall man, and was perhaps rather more inclined to corpulence than became his height, which was five feet five : and he had a little round abdominal protuberance which an inch and a half added to his heels hardly enabled him to carry off. This gave him an air of not being entirely at ease. There was however a personal dignity in his demeanour, a propriety in his gait and an air of authority in the gestures, which should prohibit one from stigmatising his efforts as a failure. . . . These trifling defects were more than atoned for by the peculiar dignity of his countenance. If his legs were short, his face was not. His hair was grey and stood straight up off his temples : his whiskers were of admirable shape. His enemies declared that their perfect shade was produced by a leaden comb. His eyes were very effective and well under command : a pair of eyeglasses was always on his nose or in his hand. The amount of secret medical knowledge of which he could give assurance by a pressure of his lips was truly wonderful. [He calls in Sir Omicron Pie.]

[At the Duke's :] "that funny little man." "Old Bolus : was apothecary at Scarrington in the old days. I remember when old

Bolus was thought to be a very good doctor." [We meet, also, Dr. Easyman, Miss Dunstable's personal physician, and Greyson, a London apothecary.]

*Framley Parsonage* (1861) gives us a sketch of Dr. Robarts of Exeter. Dr. Thorne marries Miss Dunstable.

*The Small House at Allington* (1864). In an hour the village doctor was there and expressed the opinion that it was certainly scarlatina. Mrs. Dale, not satisfied with this, sent a boy off to Guestwick for Dr. Croft, having herself no faith in the medical reputation of the apothecary. [Dr. Croft plays a considerable part in the book, but never " comes to life."]

*The Last Chronicle of Barset* (1867) shows us Dr. Turner and Balsam the apothecary, Dr. Thorne, Dr. Fillgrave, Mr. Rerechild and Sir Omicron Pie reappear.

By 1870 we have come down to times almost within living memory, and medical practice as we know it today is only just round the corner. The Medical Act, by enabling the public to distinguish the properly qualified practitioner, is eliminating the man who has passed no examinations, changes in teaching render apprenticeship obsolete and antisepsis is about to revolutionise surgery.

In Fielding we meet surgeons and barber-surgeons : Smollett shows us apothecaries, quacks, an examination at Surgeons' Hall, apprenticeship, and the relations between physicians and other practitioners. Consultants and accoucheurs are distinct classes. We see next a number of country apothecaries, gradually giving place to better-class men, till we find men of good family such as Lydgate and Thorne in general practice in the country : and London consultants with knighthoods. In the nineteenth century the apothecary has given place to the man with at least some hospital training and the diploma of M.R.C.S. is often mentioned : anæsthesia and lady doctors come into the picture.

Among all the doctors in English fiction there is one so pre-eminent that he demands at least a mention, though outside our period. For he alone was for some years famous, his name literally a household word all over Europe, America and about half Asia and Africa—I refer, of course, to Dr. Watson.

### DOCTORS INDEX

(The numbers refer to pages in text)

### Real

Allen, Dr. B., p. 98
Akenside, p. 94
Burton, p. 95
Elliotson, p. 100
Goldsmith, p. 94
Gordon, p. 93
Harison, E., p. 97
Hastings, Sir C., p. 91
Hunter, W., p. 93
Lamert, p. 98
Linden, D. W., p. 95
Parkes, p. 98
Smollett, p. 93
Smellie, p. 94

### Fictitious

Allen, Ben, p. 98
Atkins, p. 94
Badger, Mr. B., p. 99
Balsam, p. 104
Bates, p. 100
Benjamin, Little, p. 93
Best, Sir W., p. 102
Blifil, p. 93
Bold, p. 102
Bolus, p. 103
Carstone, p. 100
Carter, p. 100
Century, p. 103
Chalmers, p. 102
Chillip, p. 99
Clump, p. 101
Crab, p. 94
Croft, p. 104
Easyman, p. 104
Eskell, p. 102
Fathom, p. 94
Ferret, p. 95
Fillet, p. 94
Fillgrave, p. 103
Firmin, p. 101
Gambit, p. 97
Goddard, p. 93
Goodenough, p. 100
Greyson, p. 104
Grieve, p. 94
Gulliver, p. 92
Gunter, p. 98
Harris, p. 96
Hicks, p. 97
Hoggins, p. 101
Holt, F., p. 97
Hopkins, p. 98
Huxters, The, p. 101
Jobling, p. 99
Kenyon, p. 102
Kimble, p. 96
Lavement, p. 94
Lewsome, p. 99
Linton, p. 101
Lloyd, p. 100
Losberne, p. 99
Lumbey, p. 99
Lydgate, p. 97
Lyster, p. 95
Mackshane, p. 94
Mello, p. 100
Mew-New, Sir L., p. 103
Minchin, p. 97
Morgan, p. 94
Nockemorf, p. 98
Noddy, p. 98
Oak, Sir D., p. 101
Osmond, p. 102
Partidge, p. 93
Payne, p. 98
Pendennis, p. 100
Peps, p. 99
Perry, p. 96
Pestler, p. 101
Phillips, p. 102
Pie, Sir O., p. 103
Pilgrim, p. 96
Pilkins, p. 99
Pratt, p. 96
Rerechild, p. 103
Robarts, p. 104
Sampson, p. 102
Sawyer, Bob, p. 98
Short, p. 102
Simper, p. 94
Slammer, p. 98
Slop, p. 95
Squills, p. 101
Speers, p. 102
Sprague, p. 97
Stephenson, p. 102
Swizzle, p. 100
Terry, p. 102
Thompson, p. 94
Thorne, p. 103
Toller, p. 97
Turner, p. 104
Wolf, 102
Woodcourt, p. 100
Wrench, p. 97
Wycherley, p. 102

### REFERENCES.

Griffiths, L.M., "The Reputation of Bristol Hotwells." *Bristol Med.-Chir. Jour.*, 1902, xx. p. 1.

Harsant, W. H., "Medical Bristol in the Eighteenth Century," *Bristol Med.-Chir. Jour.*, 1899, xvii. p. 297.

*History of the British Medical Association*, London, 1932.

Kingsmill, H., *Pickwick Portrait Gallery*, London, 1936.

Latimer, J., *Annals of Bristol*, three vols., Bristol, 1893.

Layton, T. B., "Dickens's Medical Students," *Guy's Hosp. Gaz.*, 1936, l. p. 553.

Matthews, *Bristol Directory for* 1793–4. Bristol, 1794.

Parker, G., "Medical Organization . . in the Seventeenth Century," *Bristol Med.-Chir. Jour.*, 1911, xxix. P. 200.

Parker, G., "The Barber-Surgeons," *Bristol Med.-Chir. Journ.*, 1912, xxx. p. 327.

Pugh, E., *The Dickens' Originals*, Foulis, London and Edinburgh, 1913.

Smollett, T., M.D., *Essay on the External Use of Water*, London, 1752.

Underwood, E. A., "Medicine in the Writings of Smollett," *Proc. R.S.M. Sect., History of Med.*, 1937, xxx. p. 25.

